# The Development of a Melt-Extruded Shellac Carrier for the Targeted Delivery of Probiotics to the Colon

**DOI:** 10.3390/pharmaceutics9040038

**Published:** 2017-09-22

**Authors:** Noel M. Gately, James E. Kennedy

**Affiliations:** 1 Materials Research Institute, Athlone Institute of Technology, Co. Westmeath N37 HD68, Ireland; james.kennedy@kastus.com; 2 Centre for Industrial Services and Design (CISD), Department of Mechanical and Polymer Engineering, School of Engineering, Athlone Institute of Technology, Co. Westmeath N37 HD68, Ireland

**Keywords:** therapeutic delivery, shellac, probiotics, melt extrusion

## Abstract

Hot melt extrusion (HME) is considered an efficient technique in developing solid molecular dispersions, and has been demonstrated to provide sustained, modified and targeted drug delivery resulting in improved bioavailability. However, most commercial enteric or pH-responsive polymers are relatively difficult to process or have high Glass Transition Temperature (Tg) values, making their use with temperature-sensitive drugs, probiotics or biologics not viable. Shellac is a natural thermoplastic, and after a review of current literature on the pharmaceutical HME process, a possible gap in the knowledge of the use of shellac to produce dosage forms by means of HME was identified. This work explores the possibility of SSB^®^ 55 pharmaceutical-grade shellac as a melt-extrudable encapsulation polymer to entrap freeze-dried probiotic powder and to determine bacterial cell viability post-processing. Well-defined strands were produced from the physical mixture of shellac and Biocare^®^ Bifidobacterium Probiotic. FTIR clarified that there are no significant interactions between the probiotic and polymer. All of the samples demonstrated less than 5% degradation over 24 h at pH of both 1.2 and 6.8. At pH 7.4, both loaded samples gave a similar dissolution trend with complete degradation achieved after 10–11 h. Following five-month storage, 57.8% reduction in viability was observed.

## 1. Introduction

The human gastrointestinal (GI) microflora or microbiota is a complex community of microorganisms comprising of up to 500 bacterial species with approximately two million genes, known as the microbiome [1]. It is estimated that between 1000 and 1150 dominant bacterial species largely remain confined to the distal gut (colon). However, due to peristalsis movements and the antimicrobial effects of gastric acidity, the stomach and proximal small intestine may contain small numbers of bacteria in healthy individuals.

Of all the functional gastrointestinal disorders, irritable bowel syndrome has received the most interest in terms of the role of microflora in pathogenesis and of probiotics in therapy [2]. Irritable bowel syndrome (IBS) is a relatively common gastroenterological disorder predominantly dominated by symptoms such as abdominal pain, diarrhea or constipation, and bloating. The exact pathophysiology is uncertain, but possible mechanisms involve altered gut motility, visceral hypersensitivity and exaggerated stress response [3]. Probiotics seem to improve IBS symptoms and quality of life [4]. *Lactobacillus* and *Bifidobacterium* species appear to improve gut-barrier function, reduce mucosal permeability and inhibit pathogen binding [5]. The actual mechanism of action of probiotics has not been clearly understood, however, documented results are those obtained from animal models and in vitro experiments [6].

Probiotics appear to affect the intraluminal environment in a positive way, producing beneficial short-chain fatty acids and deconjugating bile acids, while inhibiting the growth of pathogenic bacteria by direct competition. Studies have reported that probiotics exert potent anti-inflammatory effects, modulating cytokine expression by interacting with gut-associated lymphoid tissue, helping to decrease the visceral hypersensitivity characteristic of IBS [7]. However, probiotic formulation manufacture poses a significant challenge, due in some part to the limited number of excipients approved for use in such therapies and diminished cell stability during the manufacturing process and storage. The successful oral delivery of probiotics is impeded by the decreased viability of the bacterial cells through the Gastrointestinal Tract (GIT) and consequent loss of survival under the effect of high acidity and bile salt concentrations [8]. Thus, an oral colon-specific delivery device derived from a natural source would be of interest for this application.

Once the probiotic bacteria reach and proliferate in the colonic region of the digestive tract, evidence of their therapeutic effects on the IBS symptoms have been reported [9]. For efficient delivery of the probiotics to the desired target site, and to allow them to manifest their positive effect in the intestinal tract, some specific necessary criteria should be fulfilled. Firstly, probiotics should survive the manufacturing process and storage in order to be viable in the final commercial product at the end of the shelf life, above a threshold of 10^6^ CFU/g (CFU, Colony Forming Unit); Secondly, the bacteria should be protected and resist the harsh physicochemical conditions in the gastrointestinal tract [10,11]. The extreme acidity in the stomach, surfactant bile acids in intestinal fluids and assorted digestive enzymes along the gastrointestinal tract (GIT) are the major obstacles to circumvent for successful probiotic delivery [9]. The gastric pH of the stomach of a healthy human adult in the fasting state is reported as approximately 1.5, which can elevate to between 3.0 and 5.0 during feeding [1]. Bifidobacteria are common probiotic microorganisms, with the most recognized species being *Bifidobacterium adolescentis*, *Bifidobacterium animalis*, *Bifidobacterium bifidum*, *Bifidobacterium breve*, *Bifidobacterium infantis*, *Bifidobacterium lactis* and *Bifidobacterium longum*. They are gram-positive, rod-shaped bacteria that are strictly anaerobic. These bacteria can grow at pH in the range 4.5–8.5, and actively ferment carbohydrates, producing mainly acetic acid and lactic acid in a volume ratio of 3:2 (*v*/*v*), but not carbon dioxide, butyric acid or propionic acid [1].

Colon-targeted oral delivery for the treatment of colon-related diseases is considered to be the best delivery strategy, as it allows for specific drug administration to the diseased site, improved drug bioavailability and better patient compliance [12]. In relation to delivery systems to the colon, the system needs to release the therapy that has close-to-neutral pH [13]. Shellac is reported to have a reasonably high dissolution pH of about 7.3 [14], and this characteristic qualifies shellac for application in colon-targeting formulations [15]. The addition of excipients is often necessary to accelerate drug release in the small intestine. When shellac is utilized as a coating layer, the formulation usually is resistant to dissolution in the stomach until it reaches gut regions with higher pH, mainly the distal ileum along with the transverse and descending colon [16]. This provides an opportunity to allow the transport of drugs into the colon for a localized treatment of the colonic ailments. Furthermore, some sustained-release formulations have also been developed. Pearnchob et al. [17] investigated drug release from shellac-containing matrix tablets prepared by either compression of powder or granules, which provided sustained drug release depending on the drug/shellac ratio. In more recent work, enteric properties of shellac were improved and probiotic formulations were developed comprising this natural polymer [11]. Recently, a study to evaluate the effect of 5-fluorouracil (5-FU) release from a guar gum matrix tablet upon addition of probiotics, and shellac coating for development of an efficient colon-specific drug delivery system, were reported [18]. The authors concluded that probiotic matrix tablets coated with 10% shellac solution had potential for successful colon-specific delivery.

High temperatures during any manufacturing process have the potential to denature many pharmaceutical actives such as probiotics, protein-based actives and heat-sensitive active pharmaceutical ingredients (APIs). Low-temperature melt extrusion, which, as the name suggests, employs temperatures well below 100 °C, could offer a novel manufacturing process for probiotic formulations. Following a widespread review of available literature, the authors were unable to ascertain any published work whereby probiotics were exposed to the extrusion process and subsequent viability assessed. This work will explore the suitability of SSB^®^ 55 pharmaceutical-grade shellac as a melt-extrudable encapsulation polymer to entrap the freeze-dried probiotic powder and to determine bacterial cell viability post-processing.

## 2. Materials and Methods

### 2.1. Materials

The shellac used throughout this work was SSB^®^ 55 pharmaceutical grade, which was purchased from SSB Stroever GmbH, Bremen, Germany. The BioCare Bifidobacterium bifidum (INT B2) was purchased Biocare (Birmingham, UK). Bifidus Selective Medium Agar (Fluka 88517) and BSM supplement (Fluka 83055) were both sourced from Sigma Aldrich (Wicklow, Ireland). All materials were used as received.

### 2.2. Compounding

**Figure 1:**
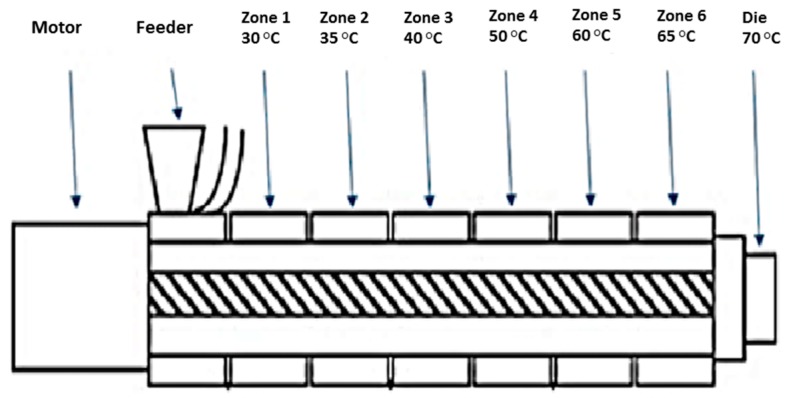
Schematic illustration of the APV twin-screw extruder and extrusion conditions used to compound SSB^®^ 55 pharmaceutical-grade shellac.

Melt compounding was carried out on a bench-top APV twin-screw extruder (MP19 TC25, APV Baker, Newcastle-under-Lyme, UK) with 19 mm diameter screws and a 35/1 length-to-diameter ratio. APV co-rotating extruder screws are designed and manufactured in a modular construction. The screws were configured of individual sections that slide onto a keyed or splined shaft. Therefore, different screw configurations using narrow-disk bi-lobal kneading elements can be arranged at any location along the shaft to generate controlled shear or mixing effects. The material to be compounded was hand-mixed in a sealed container by shaking vigorously for 2 min and subsequently, fed at a constant rate into the hopper of the APV twin-screw extruder by means of a screw-feed system. The speed of the delivery screws was maintained at such a rate as to ensure that the materials were starve-fed into the mixing screws. This ensured that in all cases output was independent of screw speed. Samples were prepared with 25% (*w*/*w*) and 50% (*w*/*w*) probiotic powder, premixed with SSB^®^ 55 shellac and starve-fed into the APV twin-screw extruder. The screw speed was fixed at 100 rpm and the heating zones were set and allowed to equilibrate. Extrusion samples were prepared using the extrusion profile outlined in Figure 1. The residence time of the materials in the extruder was in the range of 1–3 min. A circular die with an opening of 2 mm diameter was mounted to the extruder. The extrudates were air-cooled to between 30 and 50 °C before sample preparation. Prior to extrusion, the shellac material was dried using a fan oven at 40 °C for 24 h.

### 2.3. Differential Scanning Calorimetry (DSC)

To examine the thermal transition characteristics of the formulations, a TA Instruments 2010 DSC (TA Instruments, Herts, UK) was utilized. Samples were prepared by manually cutting a small sample, weighing (9–12 mg) using Sartorius scales having a resolution of 1 × 10^−5^ g and inputting the specific weight into the TA software for accurate measurement. Samples were then placed in non-perforated aluminium pans, which were crimped before testing, with an empty crimped aluminium pan being used as the reference cell. Volatiles were removed from the purging head with nitrogen at a rate of 30 mL/min. Calorimetry scans were carried out over varying temperature ranges between −50 and 250 °C. Calibration of the instrument was performed using indium as standard and all samples were tested in triplicate.

### 2.4. Fourier Transform Infrared Spectroscopy (FTIR)

Attenuated total-reflectance–Fourier transform infrared spectroscopy (ATR–FTIR, Perkin Elmer, Dublin, Ireland) was performed on a Perkin Elmer Spectrum One fitted with a universal ATR sampling accessory. All data was recorded at ambient room temperature, in the spectral range of 4000–520 cm^−1^. The test uses a 16-scan-per-sample cycle and a fixed universal compression force of 80 N. Subsequent analysis was carried out using Spectrum software (Version 10.5).

### 2.5. Rheometry

In all cases throughout this study, oscillatory parallel-plate rheological measurements were carried out on samples using an Advanced Rheometer AR1000 (TA Instruments, Hüllhorst, Germany) fitted with a Peltier temperature control (set to 37 or 70 °C) and a 40 mm diameter parallel steel plate. Samples were tested in triplicate (using individual samples) and the samples were subjected to a low-strain range sweep from 1.88 × 10^−4^ to 1 × 10^−3^ at a frequency of 1 Hz, while a constant gap of 2000 µm was utilized on the samples to ensure proper contact between the polymer and the surface plates of the instrument. The shear storage (elastic) modulus G’ and the shear loss (viscous) modulus G” were noted for determining the comparative strength of the shellac.

### 2.6. Degradation Testing

Extrudate samples of constant size and surface area were produced by cutting the extrudate strands manually into 5 ± 0.5 mm long segments and placing into physiological-type buffer at 37 ± 0.5 °C in the Sotax AT7 smart dissolution system. The dissolution of shellac into solution was detected at 230 nm in order to monitor the degredation of the matrix. Tests were carried out in triplicate using the Basket method (USP XXV, United States Pharmacopeia XXV). The media used in these tests consisted of 0.2 M hydrochloric acid (pH 1.2), and manually prepared phosphate buffer solutions (pH 7.4). The stir rate was maintained at 100 rpm with 900 mL of dissolution media used per vessel. The wavelength and absorption value for 100% concentration of the shellac in media was determined using a Perkin Elmer Lambda 40 UV/Vis spectrometer (Perkin Elmer, Dublin, Ireland) and these values were inputted into the dissolution software for dissolution calculation purposes. Samples were automatically taken every 15 min, filtered and passed through a Perkin Elmer Lambda 20 UV/Vis spectrometer (Perkin Elmer, Dublin, Ireland), before being returned to the dissolution chamber. The dissolution profile was observed from a plot of time versus absorbance.

### 2.7. Enumeration of Probiotic Survivors Following Melt-Extrusion Process

500 mg of crushed sample was suspended in 100 mL of thioglycollate broth in a conical flask, and covered over with liquid paraffin to maintain anaerobic conditions. The spread-plate technique was employed to facilitate bacterial cell enumeration before and after processing, which involved spreading 100 μL aliquots and respective dilutions of microbial test liquid on nutrient agar plates followed by incubation at 37 °C in anaerobic conditions for a minimum of 24 h. As necessary, 1 mL of sample was agar plate poured to confirm complete microbial inactivation. Surviving populations were then determined by enumeration of colonies per mL of sample (CFU/mL) as outlined in Equation (1), where *N* is the mean value for the obtained colony count, df is the dilution factor and the factor 10 was used to recalculate the amount of counted colonies to a sample volume of 1 mL.

Number of colonies per mL sample or CFU/mL = *N* × 10 × df (1)

Samples were examined in duplicate and results were reported as mean CFU/mL values.

### 2.8. Preparation of BSM-Agar (Bifidus-Selective Medium Agar)

BSM-Agar was employed for the selective isolation, identification and enumeration of bifidobacteria dissolving and reconstituting from the samples. This medium, mainly used for quality control in the manufacture of dairy products, was chosen because in anaerobic conditions it is selective for *Bifidobacterium*, while *Lactobacillus* and *Streptococcus* strains are inhibited from growing. *Bifidobacterium* colonies generally are visible within 24–48 h, but sometimes growth might require up to three days, due in the most part to the highly selective conditions. Colonies manifest as pink–purple visible patches. BSM-Agar contains peptone and meat extract as sources of carbon, nitrogen, vitamins and minerals. Yeast extract supplies B-complex vitamins, which stimulate bacterial growth, and dextrose as the carbohydrate source. Sodium chloride maintains the osmotic balance and the medium contains reducing and buffering agents. Selective salts inhibit the growth of moulds, enterococci and other gram-negative bacteria. Another compound inhibits glycolysis by inactivating the glyceraldehyde-3-phosphate dehydrogenase present and important in different bacteria and fungi (also *Streptococci* sp.). Three antibiotics are the selective agents and inhibit the accompanying bacterial flora like Bacilli, Enterobacteriacea and Pseudomonads. Bifidobacteria can reduce an azo compound present in the medium, which gives the colonies a pink–purple coloration [19].

To prepare BSM-Agar, 55.5 g of BSM-Agar powder is dissolved in 1 liter water with the final pH adjusted to pH 7.4 before sterilization by autoclaving at 121 °C for 15 min. The solution was allowed to cool to 55 °C before the addition of 116 mg BSM supplement (Fluka 83055; freshly dissolved in 5 mL sterile water). The agar was mixed well and poured directly into sterile petri plates. The prepared plates can be stored at 4 °C in the dark for several weeks, as some components are photosensitive.

## 3. Results and Discussion

### 3.1. Plasticisation Effects

**Figure 2:**
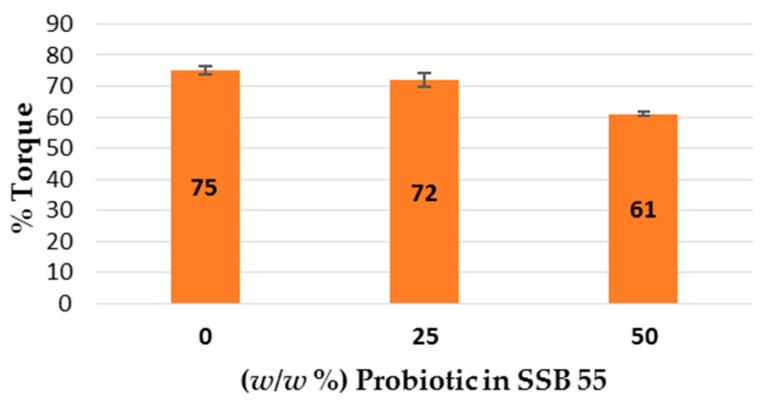
Extruder torque readings (*n* = 3) observed during processing of virgin SSB^®^ 55 pharmaceutical-grade shellac and compounded Biocare^®^ Bifidobacterium Probiotic at concentrations of 0%, 25% and 50% (*w*/*w*) in the shellac matrix.

Torque measurements observed in this study indicate shellac alone required the most energy to melt process, as can be seen in Figure 2. The high melt viscosity of shellac was reduced on addition of the Biocare^®^ Bifidobacterium Probiotic powder to the system, whereby a relatively marginal but proportional decrease in melt viscosity from 75% torque to 72% and 61% torque was observed for samples with 25% (*w*/*w*) and 50% (*w*/*w*) probiotic powder included, respectively. This indicates that the probiotic powder had a plasticizing effect within this system when compared to the virgin shellac.

### 3.2. Thermal Analysis

**Figure 3:**
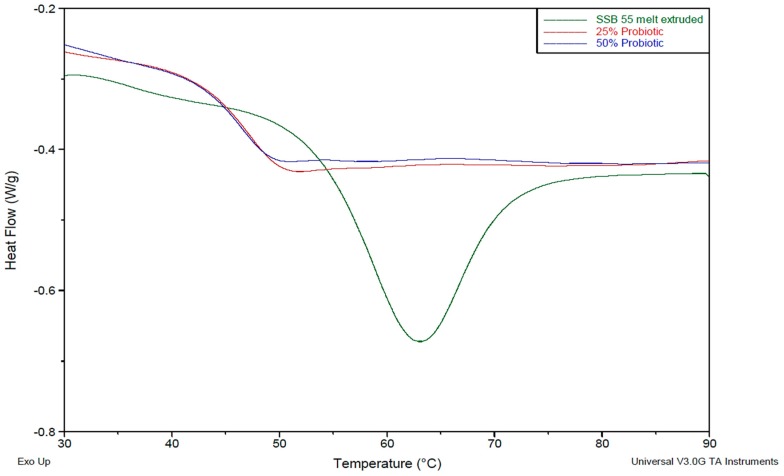
Overlaid thermograms of virgin SSB^®^ 55 pharmaceutical-grade shellac and compounded Biocare^®^ Bifidobacterium Probiotic at concentrations of 25% and 50% (*w*/*w*) in the shellac matrix.

Figure 3 illustrates that the addition of the Biocare^®^ Bifidobacterium Probiotic powder lowers the overall glass transition temperature of the material, indicating an increase in the mobility of the backbone chain. These findings correlate with the observations made during processing as previously outlined, whereby a plasticizing effect was revealed. Interestingly from the thermal analysis, the crystalline structure of the virgin shellac material has a broad melting profile. However, with the addition of the probiotic powder, the thermal profile changed to one more comparable to an amorphous material that is, by increasing the free volume. The shellac matrix in its virgin extruded form had a Tg at 58.51 °C. This transition was reduced to 49.98 and 49.51 °C upon addition of 25% and 50% probiotic powder, respectively.

### 3.3. Spectroscopy Analysis

**Figure 4:**
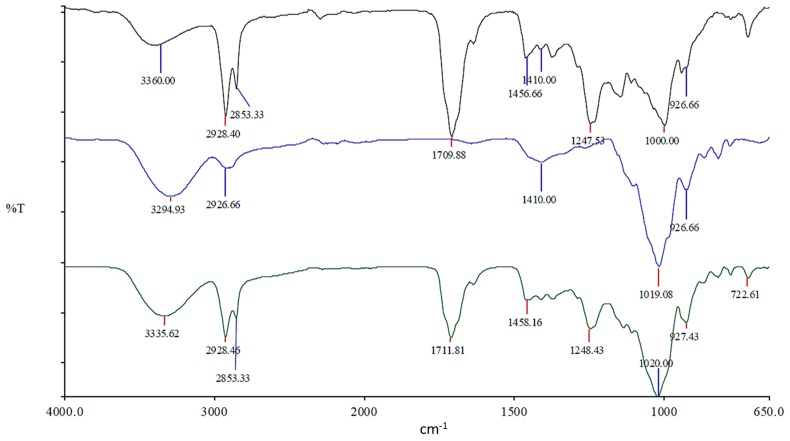
FTIR spectra for Biocare^®^ Bifidobacterium Probiotic powder (blue line–middle), 100% melt-extruded SSB^®^ 55 shellac (black line–top) and probiotic compounded with shellac at the highest loaded combined sample (50% *w*/*w*) (green line–bottom).

Figure 4 shows the FTIR spectra obtained for probiotic base powder (blue line), neat extruded SSB^®^ 55 shellac (black line) and probiotic powder compounded with SSB^®^ 55 shellac (50/50 *w*/*w* —green line) within the wavenumber range of 400 to 4000 cm^−1^. In the case of pure Biocare^®^ Bifidobacterium Probiotic powder, which contains approximately 2% (*w*/*w*) lyophilized bifidobacterial cells and 98% (*w*/*w*) fructo-oligosaccharides (FOSs), it was expected that two distinctive ranges for each component would be observed. Vodnar et al. reported on two distinctive bands which they specifically attributed to probiotic bacteria, located around 3000 cm^−1^ (~2845 and ~2929 cm^−1^), which they mainly ascribed to asymmetric stretches of methyl and methylene groups, respectively. The authors assigned these characteristics to the bacterial cell wall fatty acids. They described a fingerprint region located between 1300 and 900 cm^−1^ that is characterized by vibrational features of proteins, nucleic acids, cell membranes and cell wall components [20].

Velázquez-Martínez et al. attributed the broad absorbance band at 3600–3200 cm^−1^ to stretching of the hydroxyl groups, including carbohydrate and phenolic hydroxyl groups. The absorbance band at 3200–2800 cm^−1^ was attributed to C–H stretching and bending vibrations. The authors surmised that in the range of 1200–900 cm^−1^, a predominance of bands attributed to C–C and C–O stretching, and C–O–H and C–O–C bending—characteristic of several oligo- and polysaccharides—can be observed. Each particular carbohydrate has a specific band in this region, which is within the so-called fingerprint region where the position and the intensity of the bands are specific for each sugar, so that identification is possible [21]. Figure 4 (blue line) gives a pronounced band in the 1019 cm^−1^ range, which may attributed to the fructo-oligosaccharides present. All the characteristic peaks observed for all samples remained unchanged in the composite samples when the spectral data was superimposed with both SSB^®^ 55 shellac and the melt-extruded matrix, suggesting no chemical interaction.

### 3.4. Rheological Analysis

#### 3.4.1. Complex Viscosity versus Frequency

**Figure 5:**
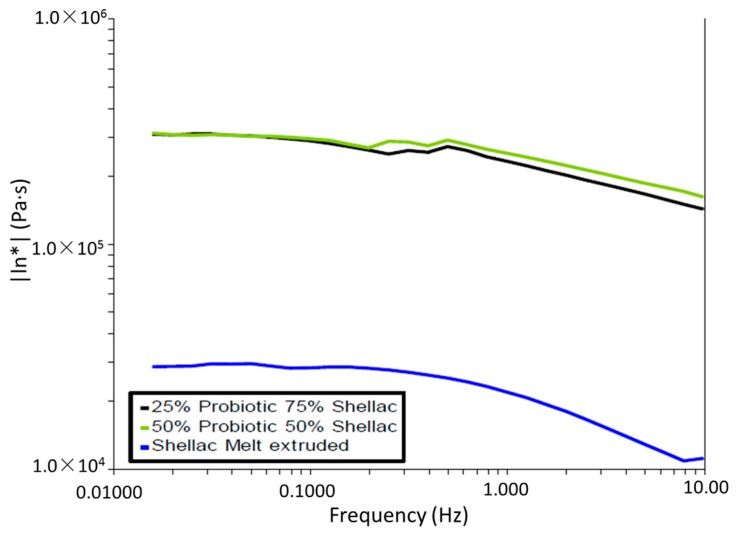
Complex viscosity versus frequency (Hz) curves at 70 °C for SSB^®^ 55 shellac and Biocare^®^ Bifidobacterium Probiotic blends.

Figure 5 illustrates the complex viscosity versus frequency (Hz) curve at 70 °C for all samples. A dramatic increase in complex viscosity is observed with the incorporation of the probiotic powder. However, there exists little change between both filled samples in terms of complex viscosity, with a similar decrease observed with increasing applied frequency across the range. With the introduction of the probiotic powder, the complex viscosity may have increased to a saturation point where the space between the polymer chains was fully occupied, reducing their mobility and increasing the polymers’ resistance to flow over the tested frequency range.

#### 3.4.2. Viscosity versus Shear Rate

**Figure 6:**
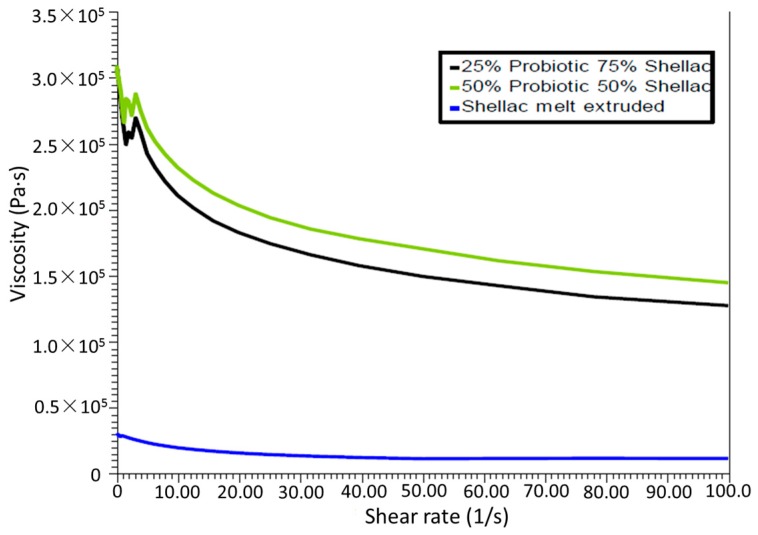
Viscosity versus shear rate at 70 °C of samples produced with SSB^®^ 55 shellac and Biocare^®^ Bifidobacterium Probiotic blends.

As presented in Figure 6, there is a higher viscosity at low shear rates for both of the filled samples, and a slight increase in viscosity is observed between the upper and lower concentrations of probiotic. All samples exhibited shear-thinning properties with the viscosity decreasing rapidly beyond a shear rate of 1 s^−1^ and a levelling off beyond a shear rate of 20 s^−1^. A more substantial increase in viscosity is obtained with the inclusion of the filler material when compared to the neat polymer, which can be explained by the presence of the high-melting-point filling material within the system.

### 3.5. In Vitro Degradation Studies

**Figure 7:**
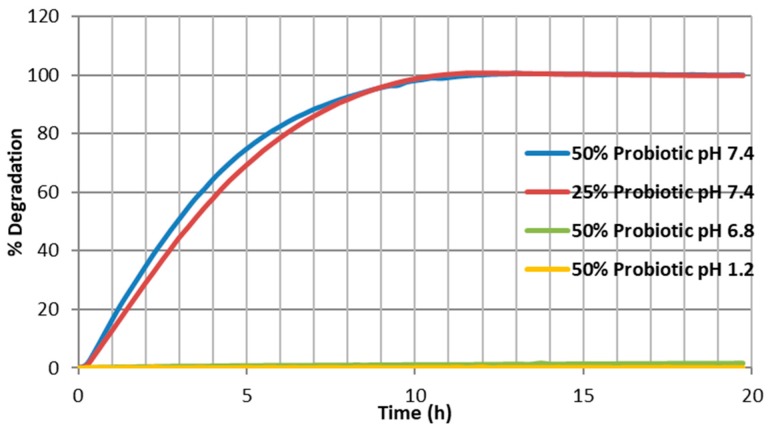
Average degradation profiles (*n* = 3) for Biocare^®^ Bifidobacterium Probiotic/shellac matrix across 3 pH ranges.

Extrudates were cut into 5 cm-long segment samples and placed into physiological-type buffer at 37 °C in the Sotax AT7 smart dissolution system (pH 7.4). The release of shellac into solution was detected at 230 nm in order to monitor the degradation of the matrix. As shown in Figure 7, both samples gave similar dissolution trends with complete degradation achieved after 10–11 h. Very little difference can be distinguished between the two samples, with only a slight variation in release shown over the first 7 h of the test, with the sample constituted with 50% probiotic powder giving a slightly quicker release of polymer. This can possibly be attributed to the fact of there being less polymer to retard the rate of breakdown in the cut extrudate sample, along with there being a higher concentration of hydrophilic and soluble probiotic powder within the sample. These two factors in combination may contribute to the quicker breakdown rate.

All of the samples demonstrated less than 5% degradation over 24 h at pH of 1.2 and 6.8, with Figure 7 demonstrating the highest loaded sample of 50% (*w*/*w*) probiotic powder, where less than 5% degradation was achieved over a 20 h period at pH 6.8. In addition, following exposure to pH 1.2 buffer, for the same period, virtually no degradation can be seen. This can be attributed to the lack of swelling capacity associated with those particular compounds and the pKa of SSB^®^ 55 shellac being about 6.11 [22], meaning SSB^®^ 55 shellac does not dissolve at pH 1.2 or pH 6.8, maintaining optimal barrier function and providing gastro–enteric resistance.

### 3.6. Growth of Bifidobacteria Cultures

The evaluation of cell viabilities in stored probiotic products is of vital importance economically and technologically, and it is important for efficacy. The survival of probiotics under unfavorable conditions, such as heating, is among the main challenges for the producers in the present market [23]. Probiotics encapsulation technologies (PETs), with the aim to protect and safely manufacture/deliver the living probiotic cell, have been explored, utilizing techniques such as spray-drying, freeze-drying, fluidized bed drying, extrusion and emulsification. Probiotic formulations are susceptible to loss in viability due to formulation, processing, storage and in vivo environment. The development of a standard protocol that simulates GIT conditions is a requirement in order to ascertain accurately whether the gastro/enteric-protected probiotics are released in simulated intestinal fluid (SIF) in vitro [8]. Problems in the stability of microorganisms commonly used in the food industry have been documented [24].

**Figure 8:**
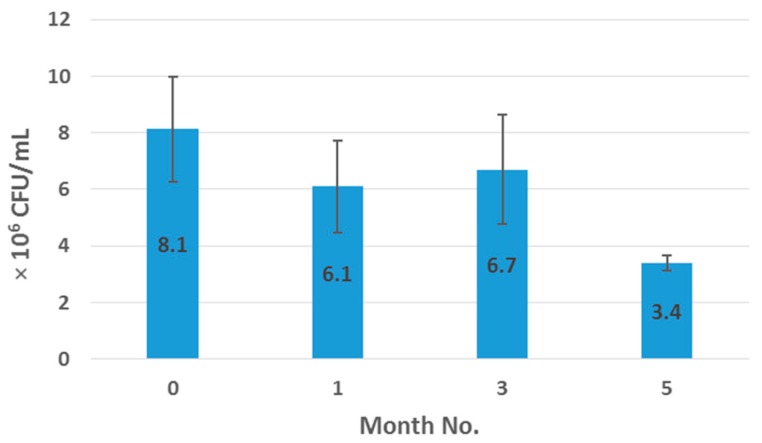
Growth of bifidobacteria from 50% sample formulation. Data points are averages from two independent fermentations.

According to manufacturer claims, each 1 g Biocare^®^ Bifidobacterium Probiotic powder should provide approximately four billion (4 × 10^9^) viable cells. Initial viable cell enumeration of neat probiotic sample yielded 1.8 × 10^8^ (*n* = 3) viable cells/g after 24 h incubation in anaerobic conditions at 37 °C. This calculates to be a reduction of 4.51% in viability when compared to the manufacturer’s claim of cell number per gram. The spread plate technique was also employed to facilitate bacterial cell enumeration before and after processing. This involved spreading 100 μL aliquots and respective dilutions of microbial test liquid on nutrient agar plates, followed by incubation at 37 °C in anaerobic conditions for a minimum of 24 h. Figure 8 shows the survival pattern of the bifidobacteria following 24 h in anaerobic test conditions over a five month period. A further 4.5% reduction in viable cell number, from 1.8 × 10^8^ CFU/mL to 8.15 × 10^6^ CFU/mL, was seen from the neat sample to the initial extrudate sample at month 0, which was enumerated 24 h after sample production. A further 25% reduction, relative to the initial processed sample, in bifidobacterial viability was observed after one-month and three-month storage in recommended dark and dry conditions at 4 °C. Following five-month storage under the same conditions, 57.8% reduction in viability to 3.4 × 10^6^ CFU/mL was observed. The cause of this reduction in viability remains unclear; however, this evaluation of probiotic survival through the melt-extrusion process was designed to be explicitly exploratory in nature. When probiotics are encapsulated, it is essential to check two conditions. First, ensure that the protective device of probiotics is reliable in media simulating the gastric fluid, and then ensure that the encapsulated probiotics are released in media simulating the intestinal fluid. In the literature, experimental models simulating the gastro–intestinal tract have been described. These models evaluate the tolerance of probiotics to acidic media, bile and enzymes [25]. However, these considerations were outside the resources and scope of this work and should be evaluated in future studies arising from this work.

## 4. Conclusions

This work explored the possibility of SSB^®^ 55 pharmaceutical-grade shellac as a melt-extrudable encapsulation polymer to entrap freeze-dried probiotic powder and to determine bacterial cell viability post-processing. Well-defined strands were produced from the physical mixture of shellac and Biocare^®^ Bifidobacterium Probiotic. Observed torque measurements indicated shellac alone required the most exertion to process, and the melt viscosity of shellac was reduced on addition of the Biocare^®^ Bifidobacterium Probiotic powder to the system. The addition of the Biocare^®^ Bifidobacterium Probiotic powder lowered the overall glass-transition temperature of the material, indicating an increase in the mobility of the backbone chain. This also indicates that the probiotic powder had a plasticizing effect within this system. When incorporated into the polymer system, plasticizers can increase the free volume between the polymer chains, which allows the chain segments to move and rotate more freely, allowing for increased movement of polymer chains with respect to each other, and consequently, decreasing the polymer Tg and altering melt viscosity. FTIR clarified that there is no significant interaction between the probiotic and polymer, and they are compatible with each other. The increase in melt viscosity became more pronounced as the percentage probiotic was increased. All of the samples demonstrated less than 5% degradation over 24 h at pH of both 1.2 and 6.8, while samples gave similar dissolution trends with complete degradation achieved after 10–11 h. Following five-month storage, 57.8% reduction in viability was observed. This suggests that a device, manufactured by the melt-extrusion process utilizing SSB^®^ 55 shellac to encapsulate probiotics to allow colon-targeted release, is a distinct possibility. However, further in-depth studies to validate this concept are required.
